# Genomic Prediction and Genome‐Wide Association Analysis of Heat Tolerance for Milk Yield in Buffaloes Using a Reaction Norm Model

**DOI:** 10.1111/jbg.70022

**Published:** 2025-10-29

**Authors:** Gabriela Stefani, Mário Luiz Santana, Lenira El Faro, Humberto Tonhati

**Affiliations:** ^1^ Departamento de Zootecnia Faculdade de Ciências Agrárias e Veterinárias, UNESP Jaboticabal SP Brazil; ^2^ Grupo de Melhoramento Animal de Mato Grosso (GMAT) Instituto de Ciências Agrárias e Tecnológicas, Universidade Federal de Rondonópolis (UFR) Rondonópolis MT Brazil; ^3^ Centro de Pesquisas de Bovinos de Corte Instituto de Zootecnia—IZ Sertãozinho SP Brazil

**Keywords:** genomic prediction, genotype by environment interaction, heat stress, random regression, temperature–humidity index

## Abstract

The aim of this study was to evaluate the impact of incorporating genomic information on the estimation of genetic (co)variance components and the accuracy of breeding values for milk yield under varying thermal environments, and to identify SNPs associated with genes that play significant roles in heat tolerance. We analysed 58,070 test‐day milk yield records from 3459 first lactations, collected between 1987 and 2018 from six herds. Genotypic data consisted of 870 animals genotyped for 45,405 SNP markers. Climatic data were obtained from INMET and combined into a temperature‐humidity index (THI). Breeding values for test‐day milk yield across THI values and days in milk were estimated using both genomic and pedigree‐based random regression animal models. The model incorporating genomic information yielded higher estimates of heritability and additive genetic variance, along with improved accuracy under heat stress conditions and better modelling of genotype‐by‐environment interaction, making it a promising approach for predicting breeding values. GWAS results were reported based on the proportion of genetic variance explained by sliding windows of five consecutive SNPs, with regions explaining more than 1% of the variance in heat tolerance selected for further consideration. The ESRRG, IGSF5 and PCP4 genes emerged as strong candidates associated with heat tolerance in milk yield.

## 1 Introduction

Buffaloes ( *Bubalus bubalis* ) are recognised for their hardiness and adaptability to hot and humid climates. According to Damasceno et al. (2010), buffaloes represent an attractive option in areas where cattle struggle to adapt, contributing to their increasing use in various regions worldwide. The buffalo population in Brazil totals approximately 1 million head (IBGE 2017), with the majority being raised on pasture under adverse climatic conditions. Although buffaloes are known for their remarkable hardiness, several studies have shown that heat stress significantly impacts the productive performance of dairy buffaloes (Choudhary and Sirohi 2019; Nasr 2017; Pawar et al. 2013; Stefani et al. 2021).

Environmental differences may contribute to variations in daughter records, leading to re‐ranking of bulls across different environments (Hammami et al. 2009; Hayes et al. 2016). This phenomenon is known as genotype by environment (G × E) interaction, where different genotypes respond differently to varying environments (Falconer and Mackay 1996). The impact of challenging environmental factors, particularly climate change and its associated heat stress responses, can result in re‐rankings of sires under different climatic conditions. Therefore, in order to remain competitive across diverse climates worldwide, international dairy cattle breeding programs should incorporate G × E interaction effects into their genetic evaluations (Ravagnolo and Misztal 2000).

Over the past decade, several studies have analysed genotype by environment interactions (G × E) for milk yield traits using random regression models (RRM) (Hammami et al. 2013; Carabaño et al. 2014). RRM allows for modelling the effect of a genotype as a function of time (e.g., days in milk) and environment (e.g., temperature‐humidity index; THI), enabling the detection of G × E interactions through differences in genetic (co)variance components for various combinations of DIM and THI (Bohmanova et al. 2008; Brügemann et al. 2011). According to Santana et al. (2015), G × E interactions can lead to significant selection errors between different environments, thereby hindering genetic progress for the trait. In this context, Stefani et al. (2021) studied Brazilian dairy buffaloes and found significant G × E interactions when applying a pedigree‐based random regression model to milk yield while incorporating a function for THI. Additionally, genetic variance for heat tolerance enables selection for genetically heat‐tolerant animals, which can help maintain high productivity while ensuring survival under adverse conditions (Dash et al. 2016), making it an important objective for genetic improvement programs.

Traditional selection methods, which use pedigree‐based relationship matrices, can achieve long‐term genetic gain. However, traditional pedigree‐based selection methods are characterised by a slower rate of genetic progress due to reduced accuracy, since they cannot capture Mendelian sampling variation when animals lack phenotypic records and predictions rely solely on parental information, as well as due to longer generation intervals (Goddard et al. 2011; Brito 2021). Furthermore, given that progeny testing is one of the most costly and time‐consuming phases of a breeding program, there is a need to develop more efficient strategies to model G × E interactions and improve prediction accuracy (Morais Júnior et al. 2018). The single‐step approach allows for the simultaneous integration of phenotypic records, genealogy and available genomic data (Aguilar et al. 2010; Christensen and Lund 2010; Legarra et al. 2009). By combining this approach with random regression models, it is possible to accelerate genetic progress, reduce generation intervals, and estimate more accurate breeding values compared to pedigree‐based analyses alone (Kang et al. 2017; Legarra and Ducrocq 2012; Zhang et al. 2016). However, to date, no studies have incorporated the single‐step approach into an RRM to model the effects of heat stress on milk production in buffaloes.

Another application of genomic random regression models (RRM) is the genetic characterisation of complex phenotypes and the identification of candidate genes in proximity to or in linkage disequilibrium with markers across environments through genome‐wide association studies (GWAS; Mota et al. 2020). The weighted single‐step GWAS is a method that allows for the estimation of SNP effects using GEBVs predicted by the single‐step approach (Wang et al. 2012). This method enables the efficient implementation of complex models, such as RRM, for genomic prediction of longitudinal traits (Kang et al. 2017) and accommodates unequal variances for SNPs, leading to improved precision in estimating SNP effects (Wang et al. 2012). Therefore, genetic progress could be accelerated by identifying the single nucleotide polymorphisms (SNPs) responsible for heat tolerance in milk yield and utilising them in marker‐assisted selection. However, there are currently no reports of candidate gene mapping studies conducted within a genomic reaction norms framework in buffaloes.

The main objectives of this study were: (1) to compare genetic (co)variance components and the prediction accuracies of breeding values for milk yield in the first lactation under different thermal environment conditions, using random regression models with and without genomic information, applying ssGBLUP and BLUP, respectively; (2) to investigate the effect of including genomic information on the genetic components related to heat tolerance in the first lactation; and (3) to identify SNP markers associated with genes that have significant effects on heat tolerance in milk yield.

## 2 Materials and Methods

### 2.1 Phenotypes

The phenotypic, genotypic and genealogical data used in this study were provided by the Department of Animal Science, Faculdade de Ciências Agrárias e Veterinárias—UNESP, Jaboticabal, SP, Brazil. After editing, a total of 53,113 test‐day milk yield records from 3179 first lactations of Murrah buffaloes, collected between 1987 and 2018, were used in the analyses. These records came from six herds located in three Brazilian states: Rio Grande do Norte (43.5%), São Paulo (42.8%) and Ceará (13.7%). The animals were predominantly raised on pasture in regions characterised by humid tropical and semi‐arid climates. The herds have been under selection for approximately 20 years, primarily focused on males, based on the national breeding value for 305‐day milk yield.

Test‐day milk yield records were obtained between Days 5 and 305 of the first lactation, with each lactation meeting at least the following five criteria: a minimum duration of 90 days; the first test‐day occurring before 75 days of lactation; at least four test‐days; the animal aged between 22 and 60 months; and the animal having at least one known parent. Contemporary groups (CG) were defined as herd‐year‐month of the test‐day and had to contain at least four animals. This definition has been widely adopted in genetic evaluations of dairy buffaloes and cattle in Brazil (Aspilcueta‐Borquis et al. 2012; Santana et al. 2015, 2017), ensuring comparability with the national evaluation system. Records with values exceeding 3.0 standard deviations above or below the CG mean were excluded. Based on the results of a preliminary analysis, six classes of days in milk (DIM) with 50‐day intervals were defined: 5–54, 55–104, 105–154, 155–204, 205–254 and 255–305 days. The pedigree file was constructed by tracing 7 generations back from the individuals with phenotypic or genotypic information, totaling 4522 animals.

### 2.2 Genotypes

After genotype quality control, a total of 45,405 single nucleotide polymorphism (SNP) data points from 870 Murrah dairy buffaloes were used in the analyses, of which 782 had phenotypic records. The genotyped animals were distributed among the three states as follows: 35.4% in São Paulo, 62.9% in Rio Grande do Norte and 1.7% in Ceará. The herds were partially connected through sires used in more than one herd and through genomic relationships among animals from different states. The animals were genotyped using the Affymetrix Axiom Buffalo genotyping chip (90 k SNP; Thermo Fisher Scientific, Wilmington, DE). The marker positions for this chip are based on the University of Adelaide water buffalo assembly map (UOA WB v. 1, Affymetrix, 2016). SNP quality control was performed using the preGSf90 program (Aguilar et al. 2010), applying the following exclusion criteria for markers: unmapped; duplicated; not autosomal; call rates below 0.90; minor allele frequency (MAF) less than 0.05; and deviation from Hardy–Weinberg equilibrium greater than 0.15. Additionally, only samples with call rates greater than 0.90 were included in the analyses.

### 2.3 Environmental Descriptor

Meteorological data were provided by the Instituto Nacional de Meteorologia (INMET, Brasília‐DF, Brazil). Climatic variables were measured at three standardised times (09:00, 15:00 and 21:00 h) daily at four weather stations located within 70 km of the farms. The station closest to each herd was designated as the reference station for that herd. Data collection procedures at the weather stations are standardised nationwide, following the recommendations of the World Meteorological Organization. The variables dry‐bulb temperature (T; in °C) and relative air humidity (RH; in %) for the same day as the test day were combined into a temperature‐humidity index (THI) using the equation described by NRC (1971):

$$\mathrm{THI}=\left(1.8\times T+32\right)-\left(0.55-0.0055\times \mathrm{UR}\right)\times \left(1.8\times T-26\right)$$

This formula was adopted because it is suited to the climatic variables available at Brazilian weather stations (T and RH). Additionally, several studies have demonstrated the effectiveness of this formula for similar studies (Bohmanova et al. 2008; Brügemann et al. 2011, 2012). The average daily THI, calculated as the mean of the six days (five days before and the test‐day itself), was assigned to each test‐day milk yield record. The selection of six days was based on the results of a preliminary analysis, which showed that the 6‐day average explained a greater portion of the variability in milk production. A similar approach was adopted by Bohmanova et al. (2008), Santana et al. (2017) and Stefani et al. (2021). The THI values were then rounded to the nearest multiple of 2, with the first class starting at THI = 60 and the last class starting at THI = 80, resulting in a total of 11 classes.

### 2.4 Statistical Analyses

The genetic parameters for test‐day milk yield across DIM and THI values were estimated using a random regression model (RRM). Fixed and random regressions on DIM were modelled using Legendre polynomials of order 3 (intercept, linear, quadratic, cubic), based on the approach used by Aspilcueta‐Borquis et al. (2012). The linear fixed and random regressions on THI were modelled using Legendre polynomials of order 1, which is equivalent to a classical reaction norm model (intercept and linear terms). Based on the results of a preliminary analysis, the residual variance was assumed to be heterogeneous across lactation (DIM: classes 1, 2–4 and 5–6) and THI values (THI: classes 60–62, 64–80), resulting in six different combinations. The random regression animal model was described as follows:

$$y={\text{FIXED}}_{k}+\sum_{n=1}^{q}\beta_{\ln}\omega_{n}\left(d\right)+\sum_{n=1}^{q}\gamma_{\ln}\omega_{n}\left(d\right)+\sum_{n=1}^{q}\delta_{\ln}\omega_{n}\left(t\right)+\sum_{n=1}^{q}\varepsilon_{\ln}\omega_{n}\left(t\right)+e_{\text{ijklm}},$$

where $y_{\text{jklmn}}$ is the mth TD milk yield record of the *l*th animal; ${\text{FIXED}}_{k}$ is the *k*th combination of fixed effects of GC (defined as above), classes of combination between milking frequency and DIM, regression coefficient for the linear and quadratic effects of cow's age at calving in months, regression coefficients for the linear, quadratic and cubic effects of DIM nested within classes of herd‐2 years of calving and regression coefficient for the linear effect of THI nested within DIM; $\beta_{\ln}$ the *n*th random regression coefficient for the additive genetic effect of animal *l* by DIM; $\gamma_{\ln}$ the *n*th random regression coefficient for the permanent environmental effect of animal *l* by DIM; $\delta_{\ln}$ the *n*th random regression coefficient for the additive genetic effect of animal *l* by THI; $\varepsilon_{\ln}$ the *n*th random regression coefficient for the permanent environmental effect of animal *l* by THI; *q* the number of regression coefficients; $\omega_{n}\left(d\right)$ the *n*th orthogonal Legendre polynomial corresponding to DIM *d*; $\omega_{n}\left(t\right)$ the *n*th orthogonal Legendre polynomial corresponding to THI *t*; and $e_{\text{ijklm}}$ the random residual effect. The (co)variance structure follows:

$$\left[\begin{array}{c} \begin{array}{c} \beta \\ \delta \\ \begin{array}{c} \gamma \\ \varepsilon \end{array} \end{array}\\ e \end{array}\right]=\left[\begin{array}{ccccc} A⊗G_{\beta } & A⊗G_{\beta \delta } & 0 & 0 & 0\\ A⊗G_{\delta \beta } & A⊗G_{\delta } & 0 & 0 & 0\\ 0 & 0 & I_{l}⊗P_{\gamma } & I_{l}⊗P_{\gamma \varepsilon } & 0\\ 0 & 0 & I_{l}⊗P_{\varepsilon \gamma } & I_{l}⊗P_{\varepsilon } & 0\\ 0 & 0 & 0 & 0 & I_{m}\sigma_{e_{o}}^{2} \end{array}\right]$$

where A is the numerator relationship matrix; $G_{\beta }$ and $G_{\delta }$ are (co)variance matrices of the random regression coefficients for additive genetic effects by DIM and THI, respectively; $G_{\beta \delta }$ and $G_{\delta \beta }$ the covariance matrices for additive genetic effects for combinations of DIM and THI; ⊗ the Kronecker product; $P_{\gamma }$ and $P_{\varepsilon }$ the (co)variance matrices of the random regression coefficients for permanent environmental effects by DIM and THI, respectively; $P_{\gamma \varepsilon }$ and $P_{\varepsilon \gamma }$ the covariance matrices for permanent environmental effects for combinations of DIM and THI; and $\sigma_{e_{o}}^{2}$ the residual variance, with *o* ranging from 1 to 6 according to the number of residual class; $I_{l}$ is an identity matrix of appropriate size for the permanent environmental effect (*l* is the number of animals with records) and $I_{m}$ an identity matrix of appropriate size for the residual (*m* is the number of TD records). In single‐step genomic best linear unbiased prediction models as developed by Aguilar et al. (2010), H is the hybrid matrix that combines the pedigree‐based numerator relationship matrix A with the genomic relationship matrix G, as developed by Aguilar et al. (2010). The inverse of *H* was defined as:

$$H^{-1}=A^{-1}+\left[\begin{array}{cc} 0 & 0\\ 0 & G^{-1}-A_{22}^{-1} \end{array}\right]$$

where: A is the numerator relationship matrix; $A_{22}$ is the pedigree relationship matrix among genotyped animals; G is the genomic relationship matrix, constructed by an iterative algorithm, that was completely described by Wang et al. (2012). These iterations increase the weights of SNPs with large effects and decrease those with small effects, essentially regressing them to the mean (Wang et al. 2014).

In the BLUP analysis, the pedigree‐based relationship matrix A was used instead of the H matrix. The analyses were conducted using the GIBBS3F90 program from the BLUPF90 software package (Misztal et al. 2002) using a Bayesian framework via Gibbs sampling. The prior distributions for all random effects were inverse Wishart distributions. The analysis consisted of a single chain of 1,000,000 cycles, with a conservative burn‐in period of 600,000 cycles and a thinning interval of 100 cycles. Thus, 4000 samples were effectively used. Convergence was determined by Geweke's diagnostic (Geweke 1991), visual inspection of the posterior chains of the parameters, and assessing the autocorrelation and effective sample size of the posterior parameter samples.

### 2.5 Estimation of Genetic Parameters

The additive genetic and permanent environmental (co)variances matrices were calculated as ${\mathrm{ФGФ}}^{\prime }$ and $ФP{Ф}^{\prime }$, respectively, where Ф was a matrix of Legendre polynomial functions for DIM or THI classes. The elements on the diagonals were additive genetic ($\sigma_{a}^{2}$) and permanent environmental ($\sigma_{\mathrm{pe}}^{2}$) variances for each DIM or THI class. The covariances between DIM i and THI j were calculated as ${Ф}_{i}G_{\beta \delta }Ф\prime_{j}$ and ${Ф}_{i}P_{\gamma \varepsilon }Ф\prime_{j}$. Consequently, the test‐day milk yield heritability for the i th DIM class within the j th THI class was:

$$h_{\left(\mathrm{ij}\right)}^{2}=\frac{\sigma_{\mathrm{a\beta}\left(\mathrm{i}\right)}^{2}+\sigma_{\mathrm{a\delta}\left(j\right)}^{2}+2\sigma_{\mathrm{a\beta \delta}\left(\mathrm{ij}\right)}}{\sigma_{\mathrm{a\beta}\left(\mathrm{i}\right)}^{2}+\sigma_{\mathrm{a\delta}\left(j\right)}^{2}+2\sigma_{\mathrm{a\beta \delta}\left(\mathrm{ij}\right)}+\sigma_{\mathrm{p\gamma}\left(i\right)}^{2}+\sigma_{\mathrm{p\varepsilon}\left(j\right)}^{2}+2\sigma_{\mathrm{p\gamma \varepsilon}\left(\mathrm{ij}\right)}+\sigma_{e}^{2}},$$

where $\sigma_{\mathrm{a\beta \delta}\left(\mathrm{ij}\right)}$ and $\sigma_{\mathrm{p\gamma \varepsilon}\left(\mathrm{ij}\right)}$ are covariances of *i*th DIMc and *j*th level of THI for additive genetic and permanent environmental effects, respectively; $\sigma_{\mathrm{a\beta}\left(\mathrm{i}\right)}^{2}$ and $\sigma_{\mathrm{a\delta}\left(j\right)}^{2}$ are the additive genetic variances of *i*th DIMc and *j*th level of THI, respectively; $\sigma_{\mathrm{p\gamma}\left(i\right)}^{2}$ and $\sigma_{\mathrm{p\varepsilon}\left(j\right)}^{2}$ are permanent environmental variances of *i*th DIMc and *j*th level of THI, respectively; and $\sigma_{e}^{2}$ is the residual variance.

### 2.6 Genomic Predictions Based on RRM

The additive genetic effect of the random regression coefficients was used to derive the genomic estimated breeding value (GEBV), for each DIM and THI. Therefore, the EBV and GEBV of an animal i were calculated as:

$$\left(G\right){\mathrm{EBV}}_{l}^{j,k}=\phi_{\left(j\right)k}{\hat{\alpha }}_{i}^{\prime }$$

where: ${\hat{\alpha }}_{i}^{\prime }$ is the vector of the orthogonal regression coefficients of animal i for additive genetic effects (coefficients corresponding to DIM and THI); $\phi_{\left(j\right)k}$ is a vector of orthogonal coefficients evaluated in the THI j and DIM k. Thus, the GEBVs for 305‐day milk yield (GEBV305) were calculated as the sum of the GEBVs from Days 5 to 305 of lactation at a THI value of interest. Accordingly, the conventional EBV based on the pedigree relationship matrix for 305‐day milk yield was EBV305.

To study the differences in thermal sensitivity and the effects of including genomic information on selection, we analysed the response pattern of EBV305 and GEBV305 across the THI scale for 30 top‐ranking bulls, selected from the total of 85 bulls with at least 10 daughters. These 30 bulls were chosen based on the highest EBV305 values under thermoneutral conditions (THI = 72) to allow a clearer graphical visualisation of re‐ranking. In addition, Pearson's correlation was calculated between the EBV305 and the GEBV305 of all bulls with at least 10 daughters (a total of 85 bulls) across the THI scale.

### 2.7 Validation of Genomic and Pedigree‐Based Predictions

To compare prediction accuracies between BLUP and ssGBLUP across the THI scale, a validation scheme was implemented using young animals. To simulate genomic selection at a very young age, when the animal's own performance data is not yet available, a reduced dataset was created by excluding the phenotypes of the younger animals (test‐days recorded from 2010). The validation population comprised the 150 youngest genotyped animals. This reduced dataset was then used to estimate EBV305 using the BLUP approach and GEBV305 using the ssGBLUP approach for the animals in the validation population, using the variance components previously estimated from the complete dataset.

To assess the prediction bias in each thermal environment, the linear regression coefficients of the GEBV305 and EBV305 for the validation animals, obtained from the reduced dataset, were compared with their EBV305 obtained from the complete dataset, for each THI. The linear regression model is given by:

$$y_{c}=1b_{0}+b_{1}{â}_{r}+e,$$

where: $y_{c}$ refers to the EBV305 of the validation population obtained by the complete dataset in a THI of interest; $b_{0}$ and $b_{1}$ refer to the intercept and linear regression coefficients; ${â}_{r}$ refers to the GEBV305 or EBV305 of the validation animals, obtained by the reduced dataset in a THI of interest; and e refers to the residue. The $b_{1}$ was used as an indicator of bias. The validation accuracy (*r*) was calculated from the coefficient of determination (*r* ^2^) of the regression equation.

### 2.8 GWAS for Heat Tolerance

The PostGSf90 software (Aguilar et al. 2014) was used to estimate SNP effects for the slope coefficient of the reaction norm, which reflects the animal's ability to respond to heat stress. Two iterations were performed to estimate the genetic variance explained by five adjacent SNP windows, aiming to identify potential genome regions with larger effects on heat stress tolerance. A threshold of 1.0% of the total genetic variance explained by each genomic window was applied to define the important genomic regions associated with the trait.

The reference genome UOA WB v. 1 was used to search for candidate genes potentially associated with the selected SNPs. The Genome Data Viewer tool of the buffalo genome, available on the National Center for Biotechnology Information (NCBI) website (http://www.ncbi.nlm.nih.gov), was used to identify the genes. Genes mapped within 200 kb upstream or downstream of the important SNPs were considered, as this range captures the regulatory and functional regions near the SNPs, though not necessarily contained within them (Peñagaricano et al. 2013).

## 3 Results and Discussion

**FIGURE 1:**
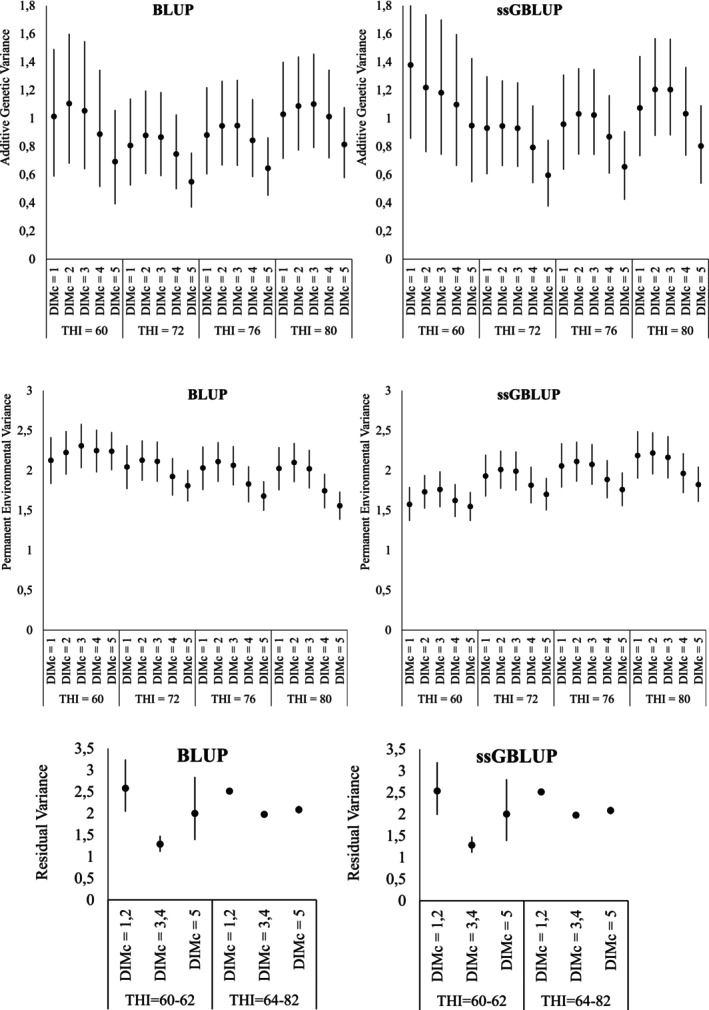
Posterior means (•) and 95% highest posterior density regions (vertical bars) of additive genetic variance (kg^2^), permanent environmental variance (kg^2^) and residual variance (kg^2^) of test‐day (TD) records for different combinations of days in milk classes (DIMc) and temperature‐humidity index (THI), estimated under BLUP (left) and ssGBLUP (right) methods.

Figure 1 presents the variance components of test‐day milk yield across DIM and THI classes, estimated by BLUP and ssGBLUP. In both analyses, considerable variation was observed along the THI scale, providing clear evidence of genotype‐by‐environment (G × E) interaction. By displaying results for all DIM × THI combinations rather than only weighted averages by THI, it became evident that these interactions are not uniform but stage‐dependent, reflecting the combined influence of thermal environment and lactation dynamics. Similar conclusions have been reported in dairy cattle, where heat stress responses are strongly modulated by lactation stage (Brügemann et al. 2011; Santana et al. 2017).

Additive genetic variance (Figure 1) followed a U‐shaped profile across the THI gradient, with lower values around thermoneutral conditions (THI ≈ 72) and higher values at both mild (THI = 60) and heat‐stress levels (THI ≥ 76). Stage‐specific patterns were also evident: at low THI, variance tended to decrease from early to mid‐lactation, whereas at high THI, variance increased toward mid‐to‐late lactation. These results suggest that genetic differences become more evident under environmental extremes, particularly when physiological demands peak, consistent with previous findings in cattle and buffaloes (Bohmanova et al. 2008; Stefani et al. 2021; Marai and Haeeb 2010). Importantly, ssGBLUP systematically increased additive genetic variance and accentuated these gradients, in agreement with studies showing that genomic information strengthens genetic links across environments and improves G × E detection (Nguyen et al. 2016; Garner et al. 2016; Hayes et al. 2016). It should also be noted that Legendre polynomials are known to inflate variance estimates at the extremes of the trajectory. Therefore, part of the higher variances observed at the beginning and end of lactation may reflect this statistical property rather than biological processes. Still, the hypothesis of stronger selection pressure in early lactation remains plausible. Future studies using non‐parametric regressors (e.g., splines) could help clarify this issue.

Permanent environmental variance (Figure 1) was higher under BLUP than under ssGBLUP, indicating that when genomic information was not included, part of the variability was absorbed by this component. By redistributing variance toward the additive genetic part, ssGBLUP reduced the relative weight of persistent non‐genetic effects, consistent with reports in dairy cattle that genomic models improve the partitioning of variance components (Misztal et al. 2009; Brügemann et al. 2011). Interestingly, BLUP suggested a gradual decrease in permanent variance with increasing THI, whereas ssGBLUP revealed a more heterogeneous profile across DIM × THI combinations, suggesting that persistent non‐genetic effects may also be modulated by environmental challenges.

Residual variances (Figure 1) showed comparable magnitudes between models, but BLUP exhibited larger oscillations across DIM classes, particularly in early lactation, and a pronounced peak under mild THI (60–62). In contrast, ssGBLUP produced more stable estimates across environments, suggesting that genomic information captured part of the heterogeneity that would otherwise inflate the residual component. Notably, residual variances did not systematically increase with THI, indicating that the models successfully allocated the effects of heat stress into additive genetic and permanent environmental components rather than into unexplained variability. Similar observations have been reported in studies where explicit modelling of THI reduced residual noise by accounting for environmental heterogeneity (Bohmanova et al. 2008; Bernabucci et al. 2014; Stefani et al. 2021).

**FIGURE 2:**
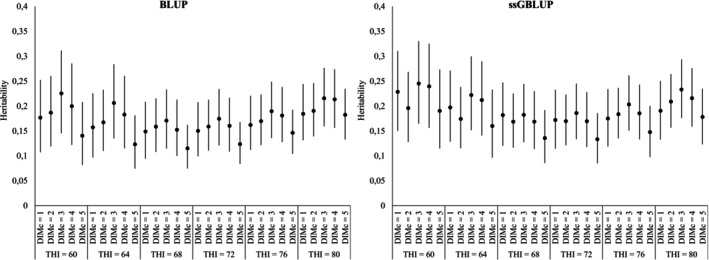
Posterior means (•) and 95% highest posterior density regions (vertical bars) of heritability estimates of test‐day (TD) records for different combinations of days in milk classes (DIMc) and temperature‐humidity index (THI) estimated under BLUP (left) and ssGBLUP (right) methods.

Figure 2 shows the heritability estimates of test‐day milk yield across DIM and THI classes. Heritability ranged from 0.12 to 0.30, indicating low to moderate genetic control of the trait. A U‐shaped profile was again observed across THI, with the lowest values around thermoneutral conditions (THI ≈ 72) and higher estimates under both mild and heat‐stress conditions. This suggests that extreme environments amplify the expression of genetic differences, whereas thermoneutral conditions—where most selection historically occurred—tend to reduce variability (Nguyen et al. 2016). Across lactation, heritability was generally higher in early stages, decreased during mid‐lactation, particularly under thermoneutral THI, and increased again toward late lactation under heat stress. These stage‐specific fluctuations highlight the combined effect of thermal load and physiological demand, as also noted in Holstein cattle (Brügemann et al. 2011; Santana et al. 2017; Carabaño et al. 2014). Finally, ssGBLUP provided slightly higher and more stable heritability estimates compared with BLUP, reinforcing the advantage of genomic information for reducing noise and improving the accuracy of genetic parameter estimation (Garner et al. 2016; Hayes et al. 2016).

**FIGURE 3:**
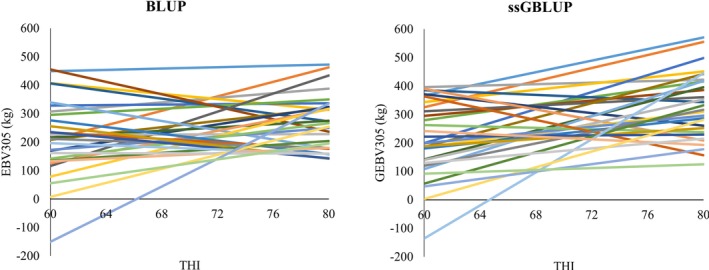
Response pattern along the temperature–humidity index (THI) scale of the 305‐day milk yield estimated breeding values (EBV305) and genomic estimated breeding values (GEBV305) for the top 30 bulls (with at least 10 daughters), ranked by EBV305 at THI = 72. [Colour figure can be viewed at wileyonlinelibrary.com]

For both ssGBLUP and BLUP methods, the slope coefficient of the reaction norm, which represents the animal's ability to respond to heat stress, exhibited greater additive genetic variance than permanent environmental variance (Table 1). This suggests that heat stress tolerance has a stronger genetic than environmental component, making it a feasible target for selection. Incorporating genomic information into the reaction norm model increased the additive genetic variance of both the intercept and slope by approximately 22% and 13%, respectively. It also resulted in lower standard deviations, indicating more precise estimates—likely due to enhanced relationship coefficient estimation. The slope/intercept ratios were similar between the two methods, indicating a meaningful G × E interaction. The genetic correlation between the intercept and slope was near zero in the BLUP method, suggesting no association between the general milk yield level and heat stress response. In contrast, ssGBLUP produced a moderate and negative correlation, indicating that animals with higher production levels tend to exhibit lower heat tolerance. This increased correlation under ssGBLUP suggests more pronounced re‐ranking of animals across different environments. These patterns were further reflected in the distribution of breeding values across THI environments, as illustrated in Figure 3.

**TABLE 1:** Posterior means and standard deviations (in parentheses) of additive genetic and permanent environmental variances for the intercept (L) and slope (S) terms of the reaction norm model estimated via ssGBLUP and BLUP approaches.

	ssGBLUP	BLUP
**Genetic additive effects**		
L	1.65 (0.24)	1.35 (0.24)
S	0.17 (0.04)	0.15 (0.04)
L–S covariance	−0.19 (0.06)	0.02 (0.06)
L–S genetic correlation	−0.35 (0.10)	0.05 (0.14)
S/L ratio	0.11 (0.03)	0.11 (0.04)
**Permanent environmental effects**		
L	2.98 (0.17)	3.32 (0.20)
S	0.01 (0.00)	0.02 (0.00)
L–S covariance	0.11 (0.01)	−0.12 (0.01)
L–S genetic correlation	0.68 (0.03)	‐0.51 (0.03)
S/L ratio	0.00 (0.00)	0.00 (0.00)

Figure 3 illustrates the response patterns of EBV305 and GEBV305 for the 30 top‐ranking bulls, evaluated across the THI scale. The varying sensitivities of animals to environmental changes influenced the distribution of breeding values in both BLUP and ssGBLUP analyses. Clear re‐rankings were observed across thermal environments, emphasising the presence of G × E interaction and highlighting the risk of selection errors if heat sensitivity is not properly modelled. The ssGBLUP approach showed more pronounced re‐ranking, which is consistent with the greater additive genetic variance observed for the slope of the reaction norm (Table 1). This reinforces the role of genomic information in capturing individual differences in heat tolerance and improving the accuracy of selection under variable climatic conditions.

**FIGURE 4:**
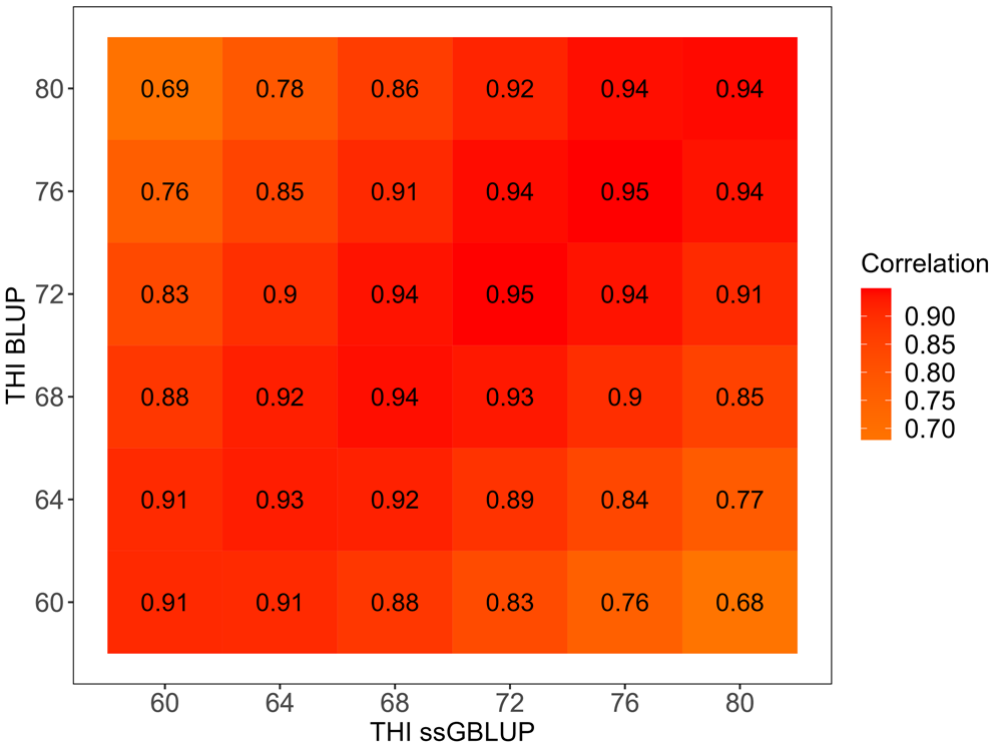
Pearson correlations between EBV305 and GEBV305 for 305‐day milk yield of 85 bulls with at least 10 daughters. [Colour figure can be viewed at wileyonlinelibrary.com]

**FIGURE 5:**
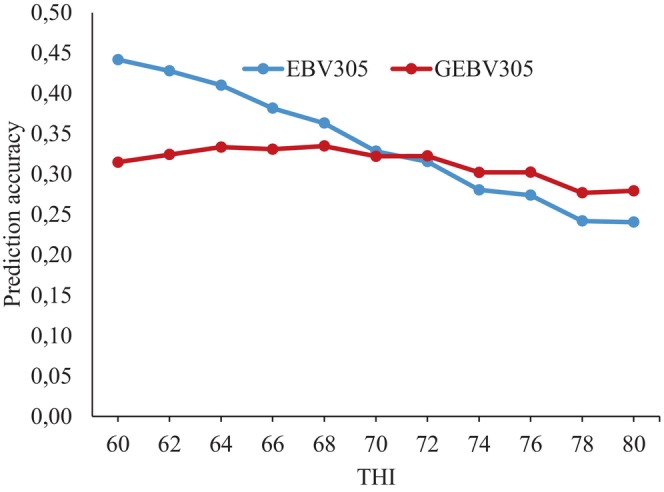
Prediction accuracies of EBV305 and GEBV305 for 305‐day milk yield across THI values. Accuracies were calculated as the correlation between breeding values predicted in the validation scheme (using the reduced dataset of the youngest genotyped animals) and those obtained from the complete dataset. [Colour figure can be viewed at wileyonlinelibrary.com]

Figure 4 presents the Pearson correlations between EBV305 and GEBV305 for the 85 most‐used sires across THI values. These correlations revealed considerable variation in genetic evaluations when genomic information was included, particularly under extreme THI conditions. Since fewer test‐day records are typically available at the extremes of the thermal scale, pedigree‐based BLUP relies more heavily on ancestral information, which limits accuracy. In contrast, ssGBLUP incorporates genomic relationships that strengthen genetic links among animals across herds and environments, thereby providing more robust predictions. This explains why the largest discrepancies between EBV and GEBV occurred at high THI values, where genomic information plays a crucial role in maintaining connectedness and reducing bias. Such findings are consistent with previous reports in dairy cattle, where genomic models improved the accuracy of breeding values in less‐represented or stressful environments (Hayes et al. 2016; Garner et al. 2016). These discrepancies highlight the environments where genomic data provide the greatest advantage, a trend that is consistent with the validation results presented in Figure 5.

Figure 5 presents the prediction accuracies obtained from the validation procedure described in the 2. Materials and Methods section, using the reduced dataset and the validation population of the 150 youngest genotyped animals. Here, accuracies refer to the correlation between breeding values predicted in the validation scheme (using the reduced dataset) and those obtained from the complete dataset. These accuracies, estimated for each THI class, allow the comparison between the BLUP and ssGBLUP approaches under different thermal environments. Overall, the prediction accuracies of EBV305 from BLUP decreased as THI increased (Figure 5). At lower THI values, accuracies appeared higher, likely because phenotypic information was scarce and the predictions from pedigree alone did not diverge substantially from those obtained using the full dataset. As THI increased, the larger amount of phenotypic information available highlighted the limitations of pedigree‐only predictions, leading to reduced accuracies. In contrast, GEBV305 accuracies remained stable across the THI scale, likely because the *H* matrix in ssGBLUP allows genomic similarity to strengthen connections between animals not related by pedigree. While BLUP generally produced higher accuracies, ssGBLUP accuracies remained more stable across THI values due to the additional genomic information strengthening connectedness, and yielded more accurate GEBVs at higher THIs (> 70). Given that 72.5% of the test‐day records in this study were collected at THIs > 70, ssGBLUP appears to be a suitable method for predicting GEBVs in these buffalo herds. These results are consistent with studies in Holsteins showing that genomic prediction models perform particularly well under challenging or less‐represented environments (Hayes et al. 2016; Morais Júnior et al. 2018).

**FIGURE 6:**
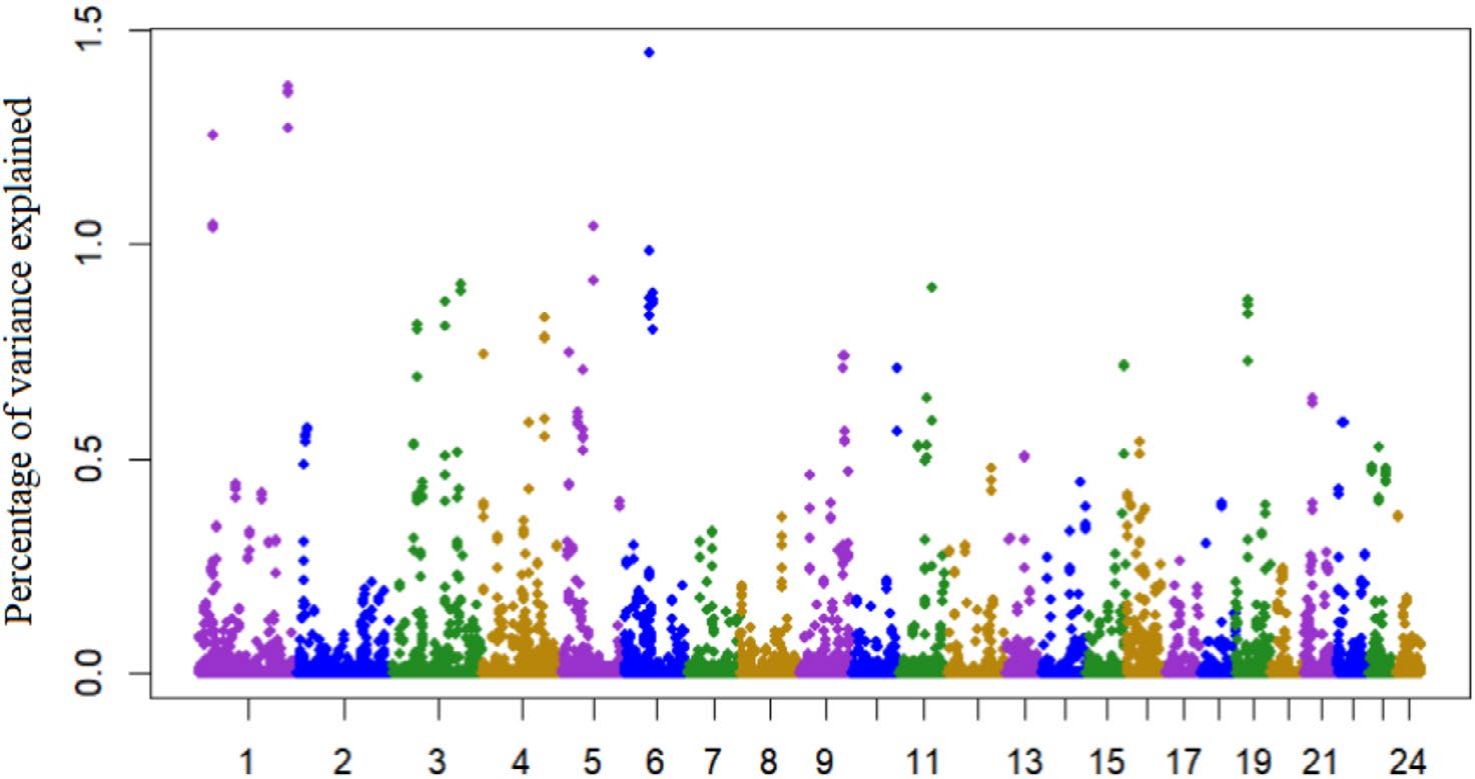
Proportion of additive genetic variance of the slope coefficient explained by 5‐SNP windows for test‐day milk yield. The x‐axis represents chromosomes (in colour), the y‐axis represents marker contributions, and the grey horizontal line marks the 1.0% threshold for candidate genomic regions. [Colour figure can be viewed at wileyonlinelibrary.com]

Figure 6 shows the results of the genome‐wide association study (GWAS) for the slope coefficient of the reaction norm, which reflects the animal's sensitivity to heat stress in milk yield. Consistent with the variance component results presented in Table 1, the GWAS highlighted that heat tolerance has a detectable genetic basis. However, most genomic windows explained less than 1% of the total additive genetic variance, underscoring the highly polygenic nature of the trait. This pattern is in agreement with previous studies in cattle, which also reported dispersed genomic signals for heat stress tolerance rather than strong major loci (Garner et al. 2016; Nguyen et al. 2016). Nevertheless, some genomic regions exceeded the 1% threshold and were selected for further investigation. The candidate genes identified within these regions are listed in Table 2 and provide valuable insights into the biological mechanisms underlying heat stress responses, complementing the quantitative genetic evidence and offering opportunities for future genomic selection strategies.

**TABLE 2:** Candidate genes mapped using NCBI database for SNPs associated with heat stress tolerance in milk yield.

ID	Símbolo	Nome	Crom.	Pos. inicial	Pos. final
102393698	ESRRG	Oestrogen‐related receptor gamma	5	60724148	61418016
112585283	LOC112585283	Not labelled	5	60829111	60866752
112585410	TRNAC‐ACA	Transfer RNA cysteine (anticodon ACA)	5	61015447	61015518
102392178	LOC102392178	Glutamic acid‐rich SH3 domain‐binding protein	1	184095392	184171535
112586660	LOC112586660	Not labelled	1	184226779	184256279
102395407	LOC102395407	Not labelled	1	184232895	184240093
102395629	B3GALT5	Beta‐1,3‐galactosyltransferase 5	1	184279283	184318251
102396557	IGSF5	Immunoglobulin superfamily member 5	1	184393961	184448054
102397063	PCP4	Purkinje cell protein 4	1	184505825	184572722
102411501	FRG1	FSHD region gene 1	1	26395523	26409563
112587455	LOC112587455	U2‐30 small nucleolar RNA	1	26401246	26401318
112587456	LOC112587456	U2‐19 small nucleolar RNA	1	26401452	26401531
112587490	LOC112587490	SNORA81 small nucleolar RNA	1	26630162	26630317
102415368	BCAR3	BCAR3 adaptor protein, NSP family member	6	49547993	49700908
112585621	LOC112585621	Not labelled	6	49693740	49701029
102415695	FNBP1L	Formalin‐binding protein 1	6	49714736	49839300
112585912	LOC112585912	U6 RNA spliceosomal	6	49753312	49753418
112585623	LOC112585623	60S ribosomal protein pseudogene L23a	6	49766701	49767207
112585622	LOC112585622	60S ribosomal protein pseudogene L9	6	49803601	49804312
112585854	LOC112585854	SNORA16B small nucleolar RNA/SNORA16A family	6	49865716	49865848
102416237	DR1	Transcriptional repressive regulator 1	6	49878865	49900876
102401316	LOC102401316	Myosin light regulatory polypeptide 9 pseudogene	6	49951657	49952552
102416561	CCDC18	Coiled‐coil domain 18	6	49978486	50090546

The genes located in the most important regions are listed in Table 2. Several of these genes are related to hormonal responses and the regulation of internal and external stimuli. ESRRG, a steroid hormone receptor, plays a crucial role in thermogenesis (Ahmadian et al. 2018). Gu et al. (2020) associated ESRRG with responses to stressful environments in native Chinese chickens. ESRRG has also been linked to milk yield in buffalo (De Camargo et al. 2015) and cattle (Venturini et al. 2014). MYL9 (LOC102401316) has been associated with panting in heat‐stressed Jersey cows (Wang et al. 2021) and immune responses to heat stress in chickens (Monson et al. 2018). SH3BGRL3 (LOC102392178) has been linked to heat‐tolerant cows (Garner 2017). IGSF5 is associated with reproductive functions and showed altered expression in heat‐stressed pregnant sows (Seibert 2018; Zhao et al. 2021). PCP4, which modulates calmodulin–calcium binding, is involved in heat stress signalling in plants (Wu and Jinn 2012) and has been linked to inflammation during heat stress in dairy cows (Shahzad et al. 2015). Despite these findings, further research is needed to elucidate the biological and functional pathways of these genes, which could provide valuable markers for genomic selection programs aiming to improve heat tolerance in buffaloes.

## 4 Conclusion

The ssGBLUP method resulted in higher estimates of heritability and additive genetic variance, along with greater accuracies under heat stress conditions. It also provided a better model for genotype‐by‐environment (G × E) interaction affecting milk production in buffaloes, making it a suitable alternative for predicting breeding values. This study is the first to include genomic information in the assessment of heat stress tolerance in buffaloes, filling a gap in the literature. Models that incorporate G × E interaction should be integrated into current selection schemes to identify animals with better heat tolerance. Several genes were identified within the candidate genomic regions, and further studies are required to explore the biological and functional pathways of these genes influencing heat stress tolerance in milk production.

## Conflicts of Interest

The authors declare no conflicts of interest.

## Data Availability

The data that support the findings of this study are available on request from the corresponding author.
